# Crystal structure of the PAS domain of the hEAG potassium channel

**DOI:** 10.1107/S2053230X16009419

**Published:** 2016-07-13

**Authors:** Xue Tang, Juan Shao, Xiaohong Qin

**Affiliations:** a State Key Laboratory of Medicinal Chemical Biology, Nankai University, Tianjin 300071, People’s Republic of China; b Department of Biochemistry and Molecular Biology, College of Life Sciences, Nankai University, Tianjin 300071, People’s Republic of China; c State Key Laboratory of Medicinal Chemical Biology, Tianjin University, Tianjin 300071, People’s Republic of China

**Keywords:** KCNH channels, PAS domain, *ether-à-go-go* channel, hEAG potassium chanels

## Abstract

KCNH voltage-gated potassium channels play critical roles in regulating cellular functions. The channel is composed of four subunits, each of which contains six transmembrane helices forming the central pore. The cytoplasmic parts of the subunits present a Per–Arnt–Sim (PAS) domain at the N-terminus and a cyclic nucleotide-binding homology domain at the C-terminus. PAS domains are conserved from prokaryotes to eukaryotes and are involved in sensing signals and cellular responses. To better understand the functional roles of PAS domains in KCNH channels, the structure of this domain from the human *ether-à-go-go* channel (hEAG channel) was determined. By comparing it with the structures of the *Homo sapiens* EAG-related gene (hERG) channel and the *Drosophila* EAG-like K^+^ (dELK) channel and analyzing the structural features of the hEAG channel, it was identified that a hydrophobic patch on the β-sheet may mediate interaction between the PAS domain and other regions of the channel to regulate its functions.

## Abstract 2

The structure of the hEAG PAS domain is presented and compared with similar segments of human and insect ion channels.

## 1. Introduction   

The *ether-à-go-go* family (KCNH) channels are voltage-gated potassium channels with important functions in the repolarization of cardiac action potential, neuronal excitability (Becchetti *et al.*, 2002 ▸), cell differentiation and tumour proliferation (Pardo & Stühmer, 2008 ▸). The KCNH family comprises EAG (*ether-à-go-go*), ERG (EAG-related gene) and ELK (EAG-like K^+^) channels (Warmke & Ganetzky, 1994 ▸).

Similar to other K^+^ channels, the members of the KCNH family are organized with four subunits surrounding a central pore (Becchetti *et al.*, 2002 ▸). Each subunit contains six transmembrane helices (S1–S6), and the opening and closing of the channels depends on the S4 helix. In the cytosolic regions, KCNH channels contain a Per–Arnt–Sim (PAS) domain at the N-terminus (Morais Cabral *et al.*, 1998 ▸) and a cyclic nucleotide-binding homology (CNBH) domain at the C-terminus (Brelidze *et al.*, 2013 ▸; Marques-Carvalho *et al.*, 2012 ▸), which has little affinity for cyclic nucleotides (Brelidze *et al.*, 2009 ▸). The cytosolic regions harbour phosphorylation sites (Wang *et al.*, 2002 ▸), with potential for interaction with kinases (Sun *et al.*, 2004 ▸), integrins (Cherubini *et al.*, 2005 ▸) and calmodulin (Schönherr *et al.*, 2000 ▸). It is proposed that these cytosolic regions can potentially regulate the channel activity and cell signalling; however, the precise functions of the cytosolic regions remain unclear.

Voltage-gated potassium channels have been associated with a number of diseases, including cancers, in previous studies. Among these channels, EAG channels have been identified to play fundamental roles because of their restricted distributions, their regulatory roles and their oncogenic and pharmacological properties (Camacho, 2006 ▸). On one hand, EAG channels have been defined in a series of cancer cells, such as prostate, colon, ovary, melanoma, liver and thyroid cancer cells (Camacho, 2006 ▸; Farias *et al.*, 2004 ▸; Meyer *et al.*, 1999 ▸; Pardo *et al.*, 2005 ▸; Ousingsawat *et al.*, 2007 ▸). EAG has been identified as a potential tumour marker (Ludwig *et al.*, 1994 ▸). In addition, various studies have associated EAG with the cell cycle and transformation (Arcangeli *et al.*, 1995 ▸). Inhibition of the EAG channel activity reduces tumour-cell proliferation, indicating its potential role as a therapeutic target (Pardo *et al.*, 2005 ▸). Despite its demonstrated role in cancers, little is known about the regulation of EAG.

Per–Arnt–Sim (PAS) domains are widespread in prokaryotes and eukaryotes (McIntosh *et al.*, 2010 ▸; Henry & Crosson, 2011 ▸). In mammals, PAS domains are involved in the regulation of cardiac rhythm, hormone secretion and kinetic activity. They either act as sensors to mediate cellular responses to environmental stimuli, such as light, ligands and action potential, or directly participate in the response processes (McIntosh *et al.*, 2010 ▸). The sensing roles of the PAS domains depend on their interaction with small molecules, for example haem, carboxylic acids and flavin mononucleotide (Möglich *et al.*, 2009 ▸; Henry & Crosson, 2011 ▸). It has been reported that some PAS domains mainly mediate protein interactions independent of ligand stimulation (Henry & Crosson, 2011 ▸). However, the definite functional roles of the PAS domains in EAG channels still need to be clarified.

In this study, we first determined the crystal structure of the PAS domain found at the N-terminus of human EAG (N-PAS domain of hEAG; PDB entry 5j7e) and compared it with the structures of *Homo sapiens* ERG (hERG) and *Drosophila* ELK (dELK). We present the structural details and discuss the implications for the functional roles of the PAS domain in the hEAG channel.

## 2. Materials and methods   

### 2.1. Protein expression and purification   

The DNA sequence encoding residues 1–146 of the N-terminus of the human EAG channel, named the N-PAS domain, was amplified from a human cDNA library by PCR. The gene was cloned into the pET-GST vector (Invitrogen), which adds a glutathione transferase (GST) tag and a PreScission protease cleavage site at the N-terminus. The reconstructed plasmids were transformed into *Escherichia coli* BL21 (DE3) cells for expression.

**Figure 1:**
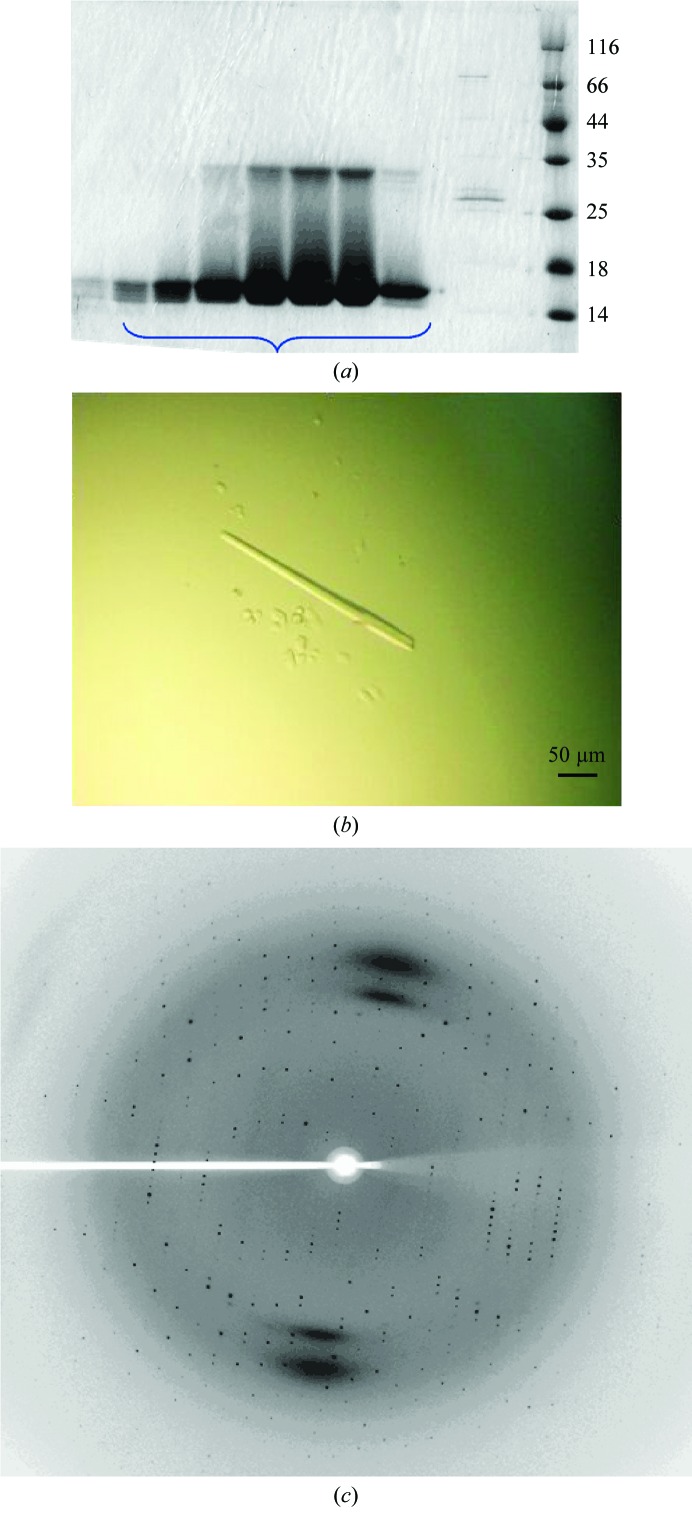
Purification, crystallization and X-ray diffraction of the N-PAS domain. (*a*) SDS–PAGE of the eluate from the HiLoad 26/60 Superdex 200 size-exclusion column. The lane on the right contains molecular-mass markers (labelled in kDa). The target protein is located at ∼17 kDa. (*b*) The crystal of the N-PAS domain used for the collection of X-ray data. (*c*) Representative diffraction image from a crystal of the hEAG N-PAS domain.

The *E. coli* cells were cultured until the OD_600_ reached ∼0.6 at 310 K and overexpression of the fusion protein was then induced using 0.2 m*M* isopropyl β-d-1-thiogalactopyranoside (IPTG) at 298 K for 16 h in LB medium (10 g l^−1^ NaCl, 10 g l^−1^ tryptone, 5 g l^−1^ yeast extract). The cells were harvested by centrifugation, resuspended in buffer *A* (20 m*M* Tris–HCl pH 7.5, 300 m*M* NaCl) and lysed by sonication. The lysate was centrifuged at 18 000*g* for 40 min and the supernatant was loaded onto a GST affinity column which had been equilibrated with buffer *A*. The column was washed with buffer *A* and eluted using 10 m*M* reduced glutathione. The fusion protein was cleaved with 2 mg ml^−1^ PreScission protease at 277 K for 16 h to remove the N-terminal GST tag. A HiTrap Q column (GE Healthcare) equilibrated with 20 m*M* Tris–HCl pH 7.5 was then used to remove the N-terminal GST tag. The column was eluted with an NaCl concentration gradient from 5 to 700 m*M*, which was applied to the column over a 75 min period, and the target protein eluted between 200 and 260 m*M* NaCl. Finally, the eluate was concentrated to 10 ml by ultrafiltration using a 10 kDa cutoff membrane. The eluate was then loaded onto a HiLoad 26/60 Superdex 200 size-exclusion column in the presence of 20 m*M* Tris–HCl pH 7.5, 200 m*M* NaCl, 5 m*M* DTT and was immediately subjected to crystallization trials. SDS–PAGE was used to determine the purity. The calculated molecular mass of the N-PAS domain is 16 933.16 Da. The eluted peak containing the N-PAS domain from the HiLoad 26/60 Superdex 200 size-exclusion column corresponded to a dimer. Samples were applied to SDS–PAGE and showed a single band at ∼17 kDa for the N-PAS domain (Fig. 1 ▸ *a*). Finally, a UV spectrophoto­meter was used to determine the concentration, and the molar extinction coefficient was calculated using *Vector NTI* (Thermo Fisher). The molar extinction coefficient was 18 260 and one *A*_280_ unit corresponds to 0.93 mg ml^−1^ protein. The sample was concentrated to 20 mg ml^−1^ for crystallization.

### 2.2. Crystallization and data collection   

Crystallization screening of the N-PAS domain was performed using the sitting-drop vapour-diffusion method. Up to 14 different series of screening solutions were prepared, including Index, Index 2, Crystal Screen, Crystal Screen 2, PEG/Ion, PEG/Ion 2, SaltRx, SaltRx 2, PEGRx and PEGRx 2 (Hampton Research, California, USA) as well as Wizard I and II (Emerald Bio). For crystallization, 1 µl protein solution (20 mg ml^−1^ N-PAS domain, 20 m*M* Tris–HCl pH 7.5, 200 m*M* NaCl, 5 m*M* DTT) was mixed with 1 µl precipitant solution (Table 1 ▸). Different combinations of precipitant, pH and salt were tested and several optimization screens were used, including Detergent Screen, Additive Screen and Silver Bullets (Hampton Research). After optimization, crystals for data collection were obtained at 291 K. The crystals were recovered and immediately flash-cooled in liquid nitrogen.

Data were collected from a single flash-cooled crystal, which was a long rod, using 25%(*v*/*v*) glycerol as a cryo­protectant on beamline BL17U at Shanghai Synchrotron Radiation Facility (SSRF; Table 2 ▸). The data were processed, integrated and scaled using *HKL*-2000 (Otwinowski & Minor, 1997 ▸).

### 2.3. Structure solution and crystallographic refinement   

The structure of the N-PAS domain was determined by molecular replacement using *Phaser* (McCoy *et al.*, 2007 ▸) from the *CCP*4 suite of programs (Winn *et al.*, 2011 ▸). The starting model was the structure of the light–oxygen–voltage-sensing (LOV) domain or PAS domain of phototropin 1 from *Arabidopsis thaliana* (PDB entry 2z6c; Nakasako *et al.*, 2008 ▸). The sequence identity between the two proteins was ∼38%, and the LOV–PAS dimer of *Arabidopsis* phototropin 1 was present in the asymmetric unit. Refinement was performed in *REFMAC*5 (Murshudov *et al.*, 2011 ▸) from the *CCP*4 suite. Model building was performed using *Coot* (Emsley *et al.*, 2010 ▸). Manual model adjustment to improve the fit to the electron-density maps was also performed using *Coot*. The stereochemistry and the agreement between the model and the X-ray data were verified using *Coot*. After the initial refinement, solvent molecules were added based on standard geometrical and chemical restraints. Residues 1–26 and 136–146 in the structure were not built in the final model because of the poor quality of the electron density. *PROCHECK* (Laskowski *et al.*, 1993 ▸) was used for validation. Details of the overall refinement and final quality of the models are shown in Table 3 ▸. Molecular comparisons were performed at PBIL (https://npsa-prabi.ibcp.fr/) and the figures were prepared using *PyMOL* (http://www.pymol.org).

## 3. Results and discussion   

### 3.1. Crystallization and optimization   

N-PAS domain microcrystals were obtained at 291 K in condition No. 54 of PEG/Ion consisting of 0.2 *M* sodium malonate pH 6.0, 20%(*v*/*v*) PEG 3350 and condition No. 88 consisting of 0.03 *M* citric acid pH 7.6, 0.07 *M* bis-tris propane pH 7.6, 20%(*v*/*v*) PEG 3350. These conditions were optimized, and after a week crystals were obtained in a condition consisting of 20%(*v*/*v*) PEG 3350, 0.1 *M* bis-tris propane pH 7.0. The crystals were suitable for X-ray analysis and diffracted to ∼3.5 Å resolution on beamline BL-17U1 at SSRF. The Additive Screen, Detergent Screen and Silver Bullets kits (Hampton Research, California, USA) were used for further optimization. Larger crystals were obtained after 7 d using condition No. 57 of the Detergent Screen kit: 20%(*v*/*v*) PEG 3350, 0.1 *M* bis-tris propane pH 7.0, 244.0 m*M* *n*-octanoyl­sucrose (Fig. 1 ▸ *c*). The N-PAS domain crystals used for X-ray diffraction were flash-cooled in liquid nitrogen with a cryoprotectant consisting of 20%(*v*/*v*) PEG 3350, 0.1 *M* bis-tris propane pH 7.0, 25%(*v*/*v*) glycerol and diffracted to ∼1.9 Å resolution on beamline 17U at SSRF (Fig. 1 ▸ *b*).

### 3.2. Structure determination of the hEAG N-PAS domain   

The crystal belonged to space group *C*2, with unit-cell parameters *a* = 213.974, *b* = 39.058, *c* = 106.802 Å, β = 118.93°. Resolution-dependent Matthews coefficient probability analysis suggested the presence of six molecules per asymmetric unit, with around 50% solvent content and a *V*_M_ of 2.54 Å^3^ Da^−1^.

### 3.3. The overall structure of the N-PAS domain of hEAG   

We have determined the structure of the PAS domain found at the N-terminus of hEAG. The three-dimensional model consisting of residues 28–137 was refined to 1.9 Å resolution, while the first 27 amino acids could not be detected in the electron-density map. Crystallographic statistics are shown in Table 1 ▸.

**Figure 2:**
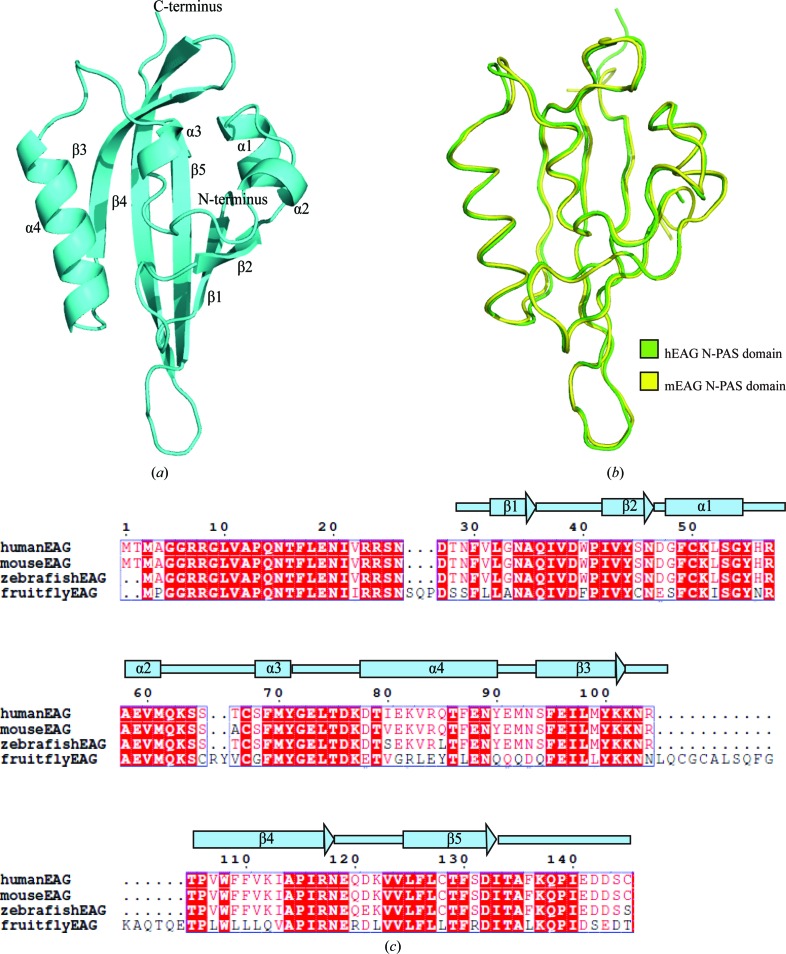
The overall structure of the PAS domain from hEAG. (*a*) Cartoon representation of the crystal structure of the hEAG N-PAS domain. Six copies are found in the crystal structure. (*b*) Superposition of PAS domains from hEAG and mEAG (PDB entry 4hoi). The hEAG N-PAS domain is shown in green and the mEAG N-PAS domain is shown in yellow. (*c*) Multiple sequence alignment and secondary structures of EAG PAS domains from human, mouse, fruit fly and zebrafish. The red boxes mark residues that are highly conserved.

In the crystal structure of the N-PAS domain, there are six molecules per asymmetric unit. Each molecule displays the canonical fold of a PAS domain comprising a central β-sheet with five strands labelled β1–β5. Four α-helices, α1–α4, decorate the β-sheet (Fig. 2 ▸ *a*). The topological order of β-strands is 2–1–5–4–3. The secondary structure from β1 to β5 is referred to as the core region of the N-PAS domain and the extensions to the N-terminus and C-terminus are referred to as flanking regions.

In order to investigate the evolutionary conservation of PAS domains in EAG channels, we aligned the amino-acid sequences of the human, mouse, fruit-fly and zebrafish proteins. The amino-acid sequences are highly conserved (Fig. 2 ▸ *c*). The crystal structure of the PAS domain from mouse EAG (mEAG; PDB entry 4hoi; Adaixo *et al.*, 2013 ▸) was superposed with the N-PAS structure through main-chain alignments and shows that apart from the N-terminal and C-terminal loops there are no obvious differences (Fig. 2 ▸ *b*). The root-mean-square deviation (r.m.s.d.) of the aligned structures is 0.362 Å. The sequence alignment shows that there are two conservative differences between the hEAG PAS domain and the mEAG PAS domain, The68 and Ile80, which were not present in either structure. These results show that the PAS-domain structures of EAG channels are highly conserved.

### 3.4. Structures of PAS domains from KCNH channels   

**Figure 3:**
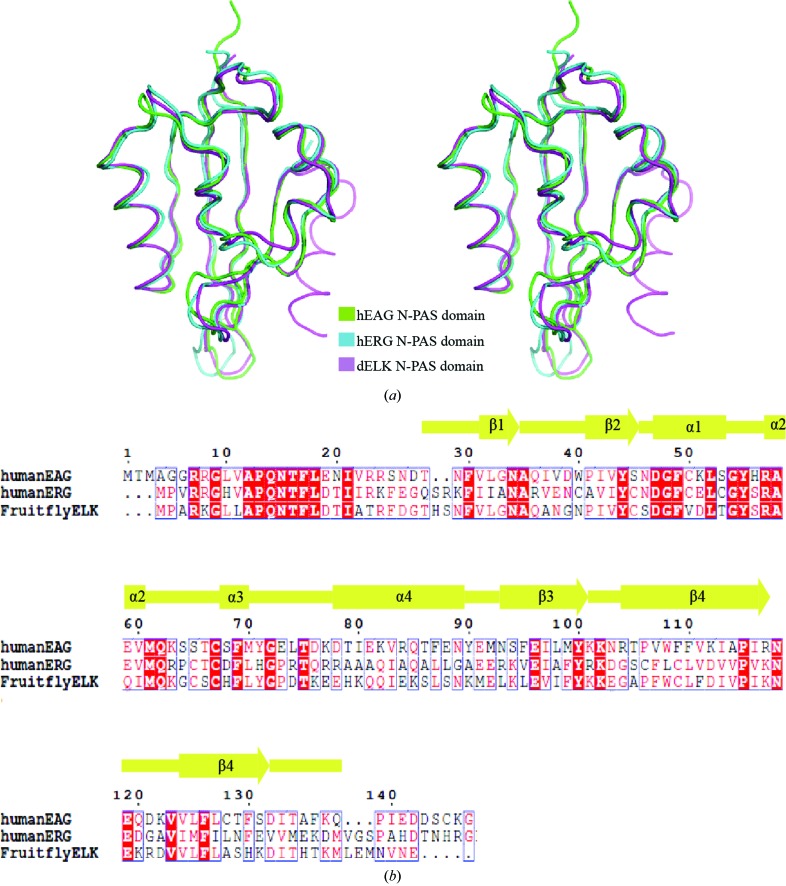
Comparison of PAS domains from hEAG, hERG and dELK. (*a*) Cross-eyed stereoview of superposition of the hEAG N-PAS domain (green) onto the hERG PAS domain (cyan) and the dELK PAS domain (magenta; PDB entry 4hp4). (*b*) Sequence alignment of PAS domains from hEAG, hERG and dELK.

To better understand the structural features of PAS domains from KCNH channels, we compared the structures of PAS domains from hEAG, mERG and dELK. We superposed these structures using their main-chain atoms. The overall structures show high similarity, apart from the N-terminal helix, which is not defined in the hEAG structure. The main differences appear in the β1–β2, α4–α5, α5–β3 and β4–β5 loop regions (Fig. 3 ▸ *a*). To analyze the differences in the amino-acid sequences, we performed a multiple sequence alignment of PAS domains from hEAG, mERG and dELK. The amino-acid sequences, including the core β-sheet region, have low conservation (Fig. 3 ▸ *b*). These results indicate that while the amino-acid sequences share little similarity among KCNH channels, the secondary structures are highly conserved.

### 3.5. Functional structures of the PAS domain of hEAG   

**Figure 4:**
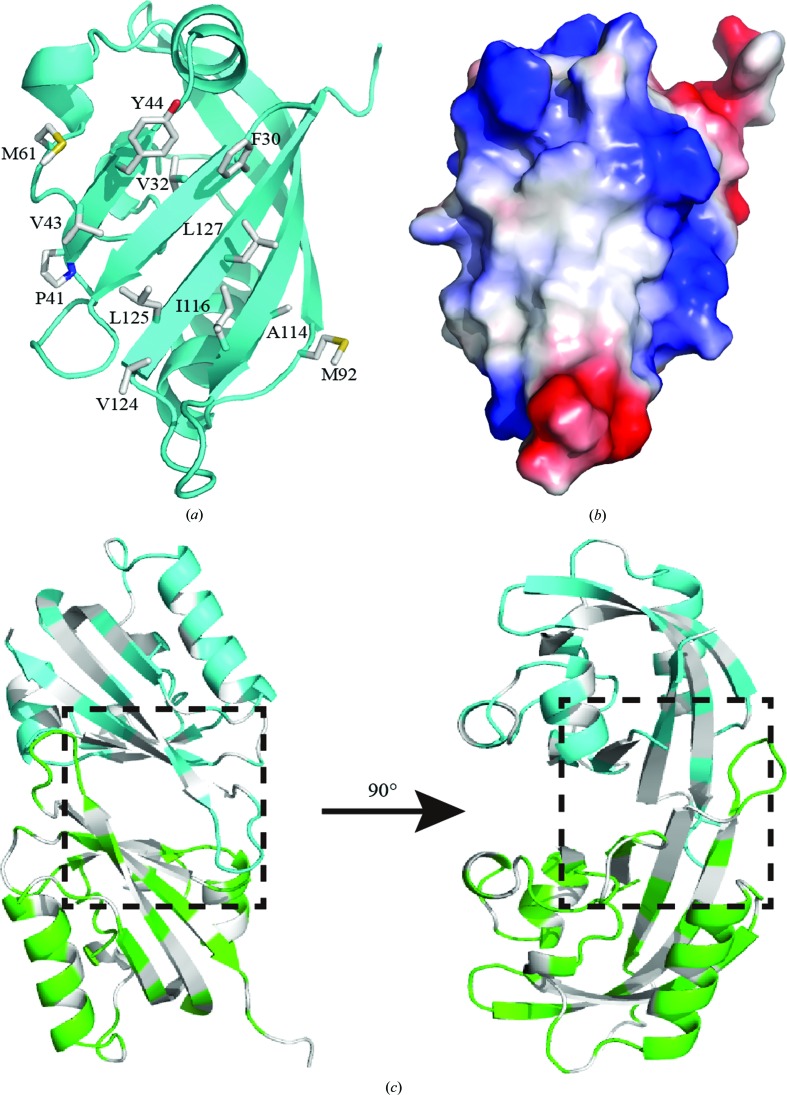
The hydrophobic patch on the outer surface of the β-sheet of the hEAG N-PAS domain. (*a*) Residues on the hydrophobic patch mediating intermolecular interaction. The side chains are shown in stick representation. (*b*) The hydrophobic patch on the surface of the structure of the hEAG N-­PAS domain. The hydrophobic region is shown in white. (*c*) Crystal contacts formed by the hydrophobic patch between molecules *A* and *D* and molecules *C* and *E*. The hydrophobic contacts are shown in white.

One of the interesting regions in the structure of the N-PAS domain of hEAG is a hydrophobic patch on the outer side of the β-sheet (Fig. 4 ▸ *b*). The patch is thought to mediate the interactions between the PAS domain and other channel regions or those among PAS domains (Gustina & Trudeau, 2011 ▸). In the ERG channel, Forster resonance energy transfer (FRET) results suggest that the PAS domain directly interacts with other parts of the channel (Gustina & Trudeau, 2009 ▸). Moreover, biochemical experiments have demonstrated direct interaction through the hydrophobic patch between a purified PAS domain and the CNBH domain (Gustina & Trudeau, 2011 ▸). The interaction is crucial for cellular functions, which serves as the molecular basis underlying long QT2 syndrome (Spector *et al.*, 1996 ▸). The hydrophobic patches in PAS domains are strongly conserved in position, size and chemical features. In the crystal structure of the N-PAS domain of hEAG, we note that the hydrophobic patches mediate interaction between molecules *C* and *E* and between molecules *A* and *D* (Fig. 4 ▸ *c*). In the hERG and dELK structures, the patches are found in the same region and also mediate the intermolecular interaction (Adaixo *et al.*, 2013 ▸). The hydrophobic patch on the hEAG N-PAS domain includes 12 apolar residues (Fig. 4 ▸ *a*) and is highly conserved in hERG and dELK. These results suggest the possibility that the functions of KCNH channels may be regulated by the interactions between this hydrophobic patch on the PAS domain and other entities. Based on the previously reported structures, the PAS domain is a monomer in hERG, while it is a dimer in dELK. It is possible that the dimerization of PAS domains may have little effect on the assembly of the channels. However, another crucial region regulating the functions of the hEAG channel, residues 1–27, at the N-terminus was not defined in the structure (Schönherr & Heinemann, 1996 ▸).

Despite many years of study, the functional roles of the PAS domain in KCNH channels still remain unclear. In particular for EAG, which has been determined as a diagnosis marker or a therapeutic target, clarification of the functional roles of the PAS domain is urgently required. In our study, we determined the structure of the PAS domain at the N-terminus of hEAG. The overall structure fits the conserved fold of the domain family. Alignment with previously determined structures of PAS domains from hERG and dELK indicates that the hydrophobic patch on the outer surface of the β-sheet may mediate both the interaction between homodimers and the interaction between the PAS domain and other channel regions in order to regulate the channel function.

## Supplementary Material

PDB reference: hEAG PAS domain, 5j7e

## Figures and Tables

**Table 1:** Crystallization

Method	Sitting-drop vapour diffusion
Plate type	48-well sitting-drop plate
Temperature (K)	291
Protein concentration (mg ml^−1^)	20
Buffer composition of protein solution	20 m*M* Tris–HCl pH 7.5, 200 m*M* NaCl, 5 m*M* DTT
Composition of reservoir solution	20%(*v*/*v*) PEG 3350, 0.1 *M* bis-tris propane pH 7.0, 25%(*v*/*v*) glycerol
Volume and ratio of drop	2 µl, 1:1
Volume of reservoir (µl)	100

**Table 2:** Data collection

Diffraction source	Synchrotron
Detector	MAR CCD, 225 mm
Wavelength (Å)	0.9796
Temperature (K)	100
Crystal-to-detector distance (mm)	150
Rotation range per image (°)	1
Total rotation range (°)	180
Exposure time per image (s)	15

**Table 3:** Data-collection and refinement statistics Values in parentheses are for the highest resolution shell.

Data-collection statistics	
Space group	*C*2
Unit-cell parameters (Å, °)	*a* = 213.974, *b* = 39.058, *c* = 106.802, β = 118.03
Wavelength (Å)	0.9796
Resolution range (Å)	50–1.90 (1.973–1.905)
No. of unique reflections	59648
Multiplicity	1.7 (1.3)
*R*_merge_ † (%)	6.8 (19.9)
Mean *I*/σ(*I*)	12.02 (4.14)
Completeness (%)	96.36 (80.22)
Refinement	
Resolution range (Å)	29.13–1.90
*R*_cryst_ ‡ (%)	21.20
*R*_free_ § (%)	22.87
R.m.s.d., bond lengths (Å)	0.006
R.m.s.d., angles (°)	1.22
Wilson *B* factor (Å^2^)	23.714
No. of non-H atoms	
Total	5744
Macromolecule	5361
Water	383
No. of protein residues	652
Residues in (%)	
Most favoured region	97.0
Additional allowed region	3.0
Generously allowed region	0
Disallowed region	0
Clashscore	13.99
Average *B* factor (Å^2^)	
Overall	28.00
Macromolecules	27.40
Solvent	36.60

† *R*_merge_ = 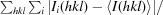 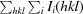.

‡ *R*_cryst_ = 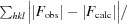 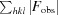.

§ *R*_free_ is calculated in the same way as *R*_cryst_ but using a test set containing 5% of the data which were excluded from the refinement calculations.
